# Preliminary Data on Free Use of Fruits and Vegetables Containing Phenylalanine 76–100 mg/100 g of Food in 16 Children with Phenylketonuria: 6 Months Follow-Up

**DOI:** 10.3390/nu15133046

**Published:** 2023-07-06

**Authors:** Alex Pinto, Anne Daly, Júlio César Rocha, Catherine Ashmore, Sharon Evans, Richard Jackson, Mary Hickson, Anita MacDonald

**Affiliations:** 1 Birmingham Women’s and Children’s Hospital, Birmingham B4 6NH, UK; alex.pinto@nhs.net (A.P.); a.daly3@nhs.net (A.D.); catherine.ashmore@nhs.net (C.A.); evanss21@me.com (S.E.); 2 School of Health Professions, Faculty of Health, University of Plymouth, Plymouth PL4 8AA, UK; mary.hickson@plymouth.ac.uk; 3 Nutrition and Metabolism, NOVA Medical School, Faculdade de Ciências Médicas, NMS, FCM, Universidade Nova de Lisboa, 1169-056 Lisboa, Portugal; rochajc@nms.unl.pt; 4 CINTESIS@RISE, Nutrition and Metabolism, NOVA Medical School, Faculdade de Ciências Médicas, NMS, FCM, Universidade NOVA de Lisboa, 1169-056 Lisboa, Portugal; 5 Reference Centre of Inherited Metabolic Diseases, Centro Hospitalar Universitário de Lisboa Central, 1169-045 Lisboa, Portugal; 6 Cancer Research UK Liverpool Cancer Trials Unit, University of Liverpool, Liverpool L69 3GL, UK; r.j.jackson@liverpool.ac.uk

**Keywords:** phenylketonuria, fruits, vegetables, metabolic control, phenylalanine

## Abstract

In phenylketonuria (PKU), a previous intervention study assessing the patients ability to tolerate fruits and vegetables containing phenylalanine 76–100 mg/100 g without limit or measurement, found that an extra 50 mg/day phenylalanine, but not 100 mg/day, was tolerated from these fruits and vegetables. In a further 6-month extension study, we examined the effect of the ‘free’ use of this group of fruits and vegetables on blood phenylalanine control. For 6 months, the patients ate fruits and vegetables containing phenylalanine 76–100 mg/100 g without limit or measurement. Three-day diet diaries and the patients’ weights were collected monthly. Blood phenylalanine spots were collected weekly aiming for blood phenylalanine levels <360 μmol/L. Retrospective blood phenylalanine was collected 6 months pre-trial. All 16 patients (69% females) from the intervention study took part in the extension study. Most of the patients (*n* = 14/16) had classical PKU with a median age of 10.5 years (range: 6–13). There was no statistically significant difference in the median blood phenylalanine pre-study (270, range: 50–760 μmol/L) compared to the 6-month extension study (250, range: 20–750 μmol/L) (*p*= 0.4867). The patients had a median of 21 and 22 bloodspots, pre- and post-trial, respectively. In the extension study, the patients had an actual mean intake of 11 g/day (4–37) natural protein and 65 g/day (60–80) protein equivalent from a protein substitute. The mean phenylalanine intake was 563 mg/day (200–1850) with only 19 mg/day (0–146) phenylalanine from fruits and vegetables containing phenylalanine 76–100 mg/100 g. The weight z-scores remained unchanged (1.52 vs. 1.60, *p* = 0.4715). There was no adverse impact on blood phenylalanine control when fruits and vegetables containing phenylalanine 76–100 mg/100 g were eaten without limit or measurement. However, the fruits and vegetable portion sizes eaten were small (60 g/week). Further longitudinal work is necessary to examine the ‘free’ use of fruits and vegetables containing phenylalanine 76–100 mg/100 g on metabolic control in patients with PKU.

## 1. Introduction

Phenylketonuria (PKU) (PKU, OMIM # 261,600) is an inherited disorder of amino acid metabolism caused by pathogenic variations in the phenylalanine hydroxylase (PAH) gene, which encodes the PAH enzyme. This affects the ability to convert the amino acid phenylalanine into tyrosine [1], causing high phenylalanine levels in the blood, brain and tissues. Untreated PKU causes severe neurocognitive dysfunction with low intelligence quotient (IQ) and developmental delay [1]. The principal treatment for PKU is a phenylalanine-restrictive diet, with the primary source of natural foods provided by low-protein fruits and vegetables. The rest of the diet consists of special low-protein foods and a protein substitute based on L-amino acids or glycomacropeptide (low/free in phenylalanine), usually supplemented with vitamins and minerals [2]. To maintain blood phenylalanine within a target range of 120 to 360 µmol/L, most patients with classical PKU tolerate ≤10 g/day natural protein (equivalent to ~500 mg/day phenylalanine) [3].

In the UK, historically, since the 1960s, fruits and vegetables containing phenylalanine <50 mg/100 g have been permitted without measurement in phenylalanine-restrictive diets. From 2003, in the PKU clinics in Birmingham, UK, all patients (including maternal PKU) have eaten fruits and vegetables (except potatoes) containing phenylalanine ≤75 mg/100 g without any adverse effect on blood phenylalanine control. This practice was introduced following the results of a carefully conducted longitudinal study in the late 1990s that showed that phenylalanine intake from fruits and vegetables containing phenylalanine ≤75 mg/100 g had a limited impact on metabolic control [4]. However, potatoes were excluded from this study. Research from two other independent European centres examining fruits and vegetables containing phenylalanine ≤75 mg/100 g demonstrated no elevation of blood phenylalanine levels over time, even though they contributed additional daily dietary phenylalanine [4,5,6,7]. Consequently, the European PKU guidelines (2017) advised that any fruits and vegetables containing phenylalanine ≤75 mg/100 g (except potatoes) could be safely given without limit, but those containing phenylalanine 76 mg/100 g or more should be calculated within the daily phenylalanine allocation [2].

There is also some evidence to suggest that patients with PKU may even tolerate unlimited use of fruits and vegetables that contain phenylalanine up to 100 mg/100 g without any impact on blood phenylalanine control. A longitudinal, uncontrolled study in Switzerland demonstrated no adverse effect on blood phenylalanine when giving unrestricted fruits and vegetables containing Phe ≤100 mg/100 g [7]. In another study, fruits and vegetables with a phenylalanine content between 76 and 100 mg/100 g were given without limit for 7 weeks, and although the minimal portion size was only 20 g (3 times weekly), the dietary phenylalanine intake was still increased by a mean of 39 mg/day but with no impact on blood phenylalanine control [4].

We performed a randomised controlled intervention trial examining the effect of increasing phenylalanine intake from fruits and vegetables containing phenylalanine 76–100 mg/100 g vs. animal sources on blood phenylalanine control in 16 children with PKU [8]. Our study showed that a consistent daily intake for 28 days of 50 mg/day phenylalanine from this group of fruits and vegetables was tolerated, but 100 mg/day of phenylalanine led to blood phenylalanine levels increasing above the recommended target therapeutic range in 31% of the patients [8]. Following this study, all the patients were invited to continue to eat fruits and vegetables containing phenylalanine 76–100 mg/100 g without measurement and incorporate these into their usual dietary practices for a further 6-month extension study. Therefore, our aim was to examine the unrestricted use of these fruits and vegetables on blood phenylalanine control in a 6-month extension study in children aged 5 to 12 years with PKU.

## 2. Materials and Methods

### 2.1. Subject Selection

Sixteen patients with PKU were studied. They attended Birmingham Children’s Hospital (UK) and were aged 5 to 12 years at the start of the randomised controlled crossover trial entitled “Impact of fruits and vegetable protein vs. milk protein on metabolic control of children with phenylketonuria: a randomised crossover controlled trial” [8]. We have previously described all the inclusion and exclusion criteria [8].

### 2.2. Study Design

**Figure 1:**
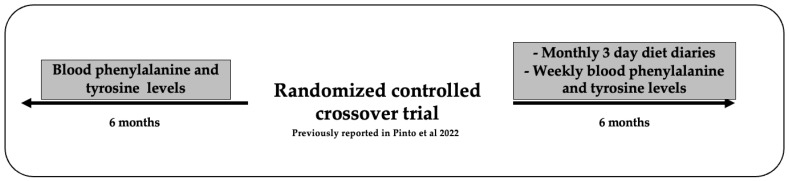
Study design of follow-up and data collected 6 months pre/post randomised control trial [8].

This was a 6-month extension study to examine the effect of fruits and vegetables containing phenylalanine 76–100 mg/100 g eaten without restriction on blood phenylalanine control following a randomised controlled crossover trial [8]. The data were collected prospectively during the follow-up extension period and retrospectively during the 6 months prior to the randomised controlled trial described in reference 8. Figure 1 shows the metabolic control data that were collected for 6 months both prior to and after the randomised controlled trial.

#### Intervention

Any fruits and vegetables containing phenylalanine 76–100 mg/100 g were eaten without measurement or limit on the portion size or frequency of consumption for the 6-month extension study. The fruits and vegetables that were given (phenylalanine content mg/100 g) were: beansprouts (92 mg/100 g), broccoli (76 mg/100 g), cauliflower (89 mg/100 g), yam (97 mg/100 g), sugar snap peas (75–88 mg/100 g), mangetout (66–93 mg/100 g), Brussels sprouts (83 mg/100 g) and figs (83 mg/100 g) [9]. It was expected that an average portion of fruits and vegetables eaten without restriction would be approximately 80 g (similar to the amounts eaten for fruits and vegetables for the general, healthy population). However, prior to the intervention study, this group of fruits and vegetables was calculated and measured within the daily phenylalanine exchange system, and approximately 60 g was equivalent to one 50 mg phenylalanine exchange.

### 2.3. Data Collection

The follow-up and retrospective data were collected between 29 January 2019 and 30 July 2021.

Trained parents/caregivers performed weekly blood spots for phenylalanine and tyrosine and posted these by first-class post to the hospital laboratory. At least two fasting blood spots were collected on a filter card (PerkinElmer Health Sciences Inc., Greenville, USA—UK Standard newborn screening) in the morning. In the laboratory, blood phenylalanine was calculated on a 3.2 mm punch by MS/MS tandem mass spectrometry. The blood phenylalanine and tyrosine levels were recorded retrospectively for 6 months pre-baseline and prospectively in the follow-up extension study. All the patients aimed to maintain blood phenylalanine control according to the PKU European Guidelines, i.e., 120 to 360 μmol/L [3]. They were advised to perform blood spots once weekly as per usual clinical practice but more frequently than European PKU guidelines [3]. If the unlimited use of fruits and vegetables containing 76–100 mg/100 g did impact on blood phenylalanine control, it was expected that over 20 blood phenylalanine spots over 6 months should identify any change in blood phenylalanine concentrations.

A weighed 3-day diet diary was completed each month by the parents/caregivers, describing the food type and portion size. The dietary intake was analysed using Nutritics^®^. The following nutrients were analysed: energy (kcal/day), carbohydrate (CHO, g/day and % energy intake), total protein (g/day, natural protein plus protein equivalent from protein substitute and % energy intake) and fat (g/day and % energy intake).

The weight measurements were performed by AP and AM using digital scales calibrated to the nearest 0.1 g (Seca GmbH Germany, Medical Measuring Systems and Scales, Birmingham, UK—Model 875). The patients’ weights were monitored monthly to check there was neither weight loss nor excessive weight gain that may have affected blood phenylalanine control. The weight z-scores were calculated according to the WHO/UK growth definitions [10,11,12].

Height was not measured as part of the study.

### 2.4. Statistical Analysis

Blood phenylalanine levels were the study’s primary outcome. A clinically relevant difference was considered to be 40 µmol/L, and a standard deviation of 40 µmol/L was used. Thirteen patients were required to obtain a statistical power of 90%. A sample size of 16 patients allowed for any dropouts. A more detailed explanation concerning the sample size calculations is given in the previous publication [8].

The statistical analysis was performed with Prism 9 (version 9.1.0), with statistical significance defined by a *p*-value <0.05. Continuous data were summarised as the median (range) or mean (SD) depending on normality testing, which was assessed by the Shapiro–Wilk test. When the distribution was normal or not normal, paired t-tests and Wilcoxon tests were performed, respectively. The categorical variables were presented as percentages.

### 2.5. Ethical Aspects

This project was provided with ethical approval by the East Midlands—Leicester South Research Ethics Committee with the reference 19/EM/0073 and Integrated Research Application System (IRAS) number 252561 and was registered on clinicaltrials.gov (ID: NCT05249218). The study followed the Good Clinical Practice guidelines and conformed to the ‘Declaration of Helsinki’ (52nd WMA General Assembly, Edinburgh, Scotland, October 2000). The parents/caregivers provided informed consent, and the patient’s gave age appropriate assent, before the study commencement.

## 3. Results

### 3.1. Participants

All the subjects with PKU (*n* = 16, 69% females—11/16) that participated in the randomised crossover trial agreed to take part and they all completed the extension study. The median age at the beginning of the follow-up period was 10.5 years (range: 6–13). Fourteen patients were of European ethnicity and two were of Asian origin. Most (*n* = 14/16) of the participants had classical PKU based on their genetic mutations or phenylalanine tolerance.

The patients’ characteristics and dietary prescriptions are described in Table 1.

### 3.2. Metabolic Control

**Figure 2:**
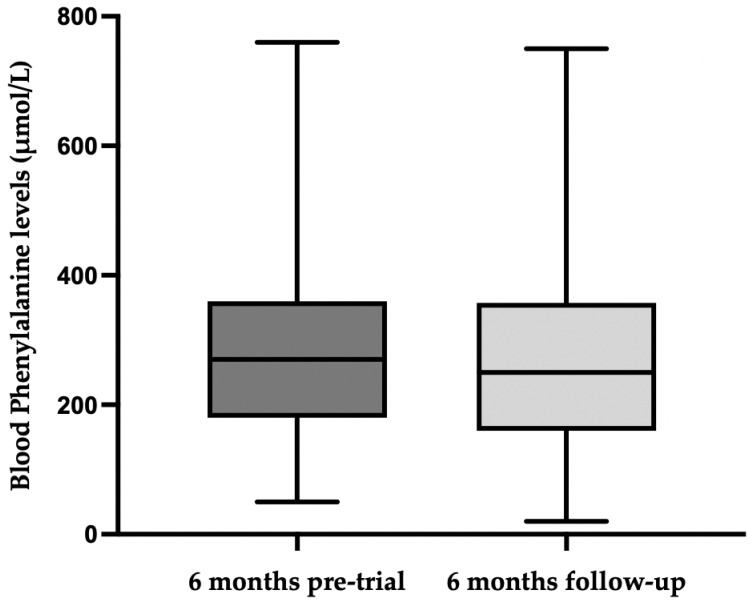
Median blood phenylalanine levels for all participants during 6-month pre-randomised controlled trial and during the 6-month follow-up.

The blood phenylalanine levels pre-baseline and during the 6-month extension study are given in Figure 2. There was no statistically significant difference in the blood phenylalanine control for 6 months pre-baseline (median, 270 μmol/L, range: 50–760) compared with the 6-month extension period (median, 250 μmol/L, range: 20–750), *p* = 0.4867.

Detailed metabolic control is presented in Table 2 for the 6 months pre-baseline and during the extension study. There were also no changes in the blood tyrosine levels. The subjects had a mean of 21 blood spots during the 6 months pre-baseline and 22 blood spots during the extension period.

### 3.3. Dietary Intake

The dietary intake remained stable during the extension study. The mean intake of protein equivalent from the protein substitute was 65 g/day (range 60–80) in the pre-randomised controlled trial and throughout the 6-month extension study. The median prescribed natural protein intake was 6 g/day (range 3–27) in the pre-randomised controlled trial and 6 g/day (range 3–27) during the extension study. Four patients were prescribed more natural protein during the extension study: subjects 1, 11 and 13 were prescribed 1 g/day extra natural protein, and the natural protein for subject 3 was increased by 2 g/day.

The actual natural protein mean intake estimated from all the foods consumed during the 6-month follow-up was 11 g/day (range 4–37). The detailed macronutrient intake data and phenylalanine intake are described in Table 3.

The fruits and vegetables containing phenylalanine 76–100 mg/100 g eaten during the extension study were broccoli (55 times), cauliflower (10 times), figs (4 times), bean sprouts (3 times) and Brussels sprouts (1 time). A mean of one portion (60 g) was eaten weekly by each child. In total, the mean phenylalanine intake from fruits and vegetables containing 76–100 mg/100 g food was 19 mg/day, ranging from no ingestion of these fruits and vegetables to a maximum of 146 mg/day of phenylalanine.

During the randomised controlled trial, four of five subjects who were unable to tolerate the 100 mg/day phenylalanine from fruits and vegetables containing 76–100 mg/100 g during the randomised control trial ate minimal amounts of these vegetables during the extension phase, except for subject 16 who often ingested one portion per day of these fruits and vegetables.

### 3.4. Participant Weight

In the extension study, there was no statistically significant change in the participant weight z-scores (1.52 vs. 1.60, *p* = 0.4715). The monthly weight z-scores are given in Table 4.

## 4. Discussion

This 6 month extension study examined the effect on blood phenylalanine control of the unrestricted use of fruits and vegetables containing phenylalanine 76–100 mg/100 g of food in a group of children with PKU. Overall, there was no change in blood phenylalanine control during the extension phase. The natural protein intake of this group of participants remained stable, although the mean daily phenylalanine intake from fruits and vegetables containing phenylalanine 76–100 mg/100 g provided an additional 19 mg/day, ranging from 0–146 mg/day. There was no change in the participants’ weight z-scores.

Even though the limited impact of fruits and vegetables containing phenylalanine 76–100 mg/100 g on blood phenylalanine control in this study is encouraging, the results need cautious interpretation. Overall, the portion sizes of the fruits and vegetables were small (mean of one 60 g serving/week), and the mean daily contribution of phenylalanine was only 19 mg. Following our randomised controlled study, after several weeks of eating the same fruits and vegetables each day, most of the children appeared disinterested in eating them regularly [8]. These results were very similar to those obtained in a previous study by MacDonald et al. [4]. The latter study included both adults and children and the unlimited use of fruits and vegetables containing phenylalanine 76–100 mg/100 g contributed an extra 39 mg/day of phenylalanine. Furthermore, a study from Switzerland was unable to demonstrate any deterioration of blood phenylalanine control when giving unrestricted intake of fruits and vegetables containing phenylalanine <100 mg/100 g [7]. Generally, children eat small portions of fruits and vegetables [13], and there is even evidence that the intake of these foods decreases during childhood [14], particularly in adolescence. The patients in our study had all been actively encouraged from the age of weaning to eat plenty of fruits and vegetables containing phenylalanine ≤ 75 mg/100 g without restriction. However, vegetables in particular were not favoured by the children. Patients with PKU commonly demonstrate neophobic eating behaviour, which may limit the variety of foods eaten [15]. Most of our children ate less than one portion per week of the fruits and vegetables we studied when they were provided in an unrestricted manner. Considering these fruits and vegetables had always been limited in their dietary regimens, this may have played a role in their food choices in the extension study. Generally, patients with classical PKU who have a low natural protein tolerance tend to eat the same foods every day and, thus, are less likely to incorporate new foods into their daily dietary routine.

In contrast, there is also concern that some patients may develop a particular taste preference for the group of fruits and vegetables studied, and, potentially, they could ingest high amounts of phenylalanine from these sources if they are given without limit. It has been established that children with PKU prefer savoury foods to sweet foods [15]. Broccoli and cauliflower containing phenylalanine between 77–100 mg/100 g are major ingredients in many popular low-protein dishes. They may be used in vegetable rice or cauliflower cheese (with low protein cheese), ‘buffalo cauliflower wings’ or ‘cauliflower popcorn.’ If eaten daily and in large portion sizes, it was clear from the randomised controlled intervention study that this would affect blood phenylalanine control. This may be a particular problem in adulthood when larger portion sizes are eaten or in pregnancy when stringent blood phenylalanine control is essential. In the 6-month extension study, some children ate an additional 146 mg/day phenylalanine from these fruits and vegetable sources, and with time, this extra phenylalanine is likely to adversely impact metabolic control in classical patients with PKU if intake becomes habitual. In contrast, patients with mild PKU or on sapropterin treatment may be able to tolerate this category of fruits and vegetables without restriction.

The most popular vegetable choice of our patients was broccoli. We did not collect detailed information about the cooking methods, but this could have affected the phenylalanine content of the vegetables at the time of consumption and, consequently, may have modified the impact on blood phenylalanine control. For example, it has been established that boiling vegetables may decrease the amino acid content by 20 to 70% when compared with the raw state. The amino acids leach out into the cooking water. Roasting or contemporary cooking methods, such as an air fryer or microwave, are less likely to result in a lower phenylalanine content. Roasting food may increase some amino acids due to the degradation of proteins and peptides into free amino acids with heat [16,17,18]. For example, the phenylalanine content of bamboo shoots was 30 mg/100 g when raw, 13 mg/100 g when boiled, 17 mg/100 g when stir-fried and 29 mg/100 g when steamed. It is also considered that cutting processes as well as the cooking method may affect cell structures [17].

Overall, the macronutrient intake was stable throughout the 6-month extension study, which reflected that the patients usually ate the same limited number of foods each day without much variability, as has been shown in previous studies [8,14]. It was important that the energy intake was maintained, as any variability may have affected the ability to tolerate any additional phenylalanine from fruits and vegetables. This was evidenced by the maintenance of weight, which followed the expected pattern, with no change in the z-scores. Overall, there was no deterioration in blood phenylalanine control, even when some patients were entering adolescence; this is a period usually associated with a decline in control [19,20,21].

### Limitations

This study had several limitations. The data on blood phenylalanine control pre-baseline were collected retrospectively. The patients did not have the same number of blood spots taken during both the pre-baseline and extension periods, although the monitoring was extensive in both parts. Although the blood phenylalanine was measured once per week during the extension period, it still may have missed the impact of the higher intake of phenylalanine associated with larger portion sizes of fruits and vegetables containing phenylalanine 76–100 mg/100 g eaten intermittently and not necessarily pre-blood sample. However, weekly blood phenylalanine spots over 26 weeks should have identified any overall change in blood phenylalanine control. There were no detailed dietary intake data collected during the 6-month pre-baseline period, so the habitual intake of fruits and vegetables containing phenylalanine 76–100 mg/100 g of food was unknown, but it would have been limited due to the requirement to calculate the phenylalanine from these sources within the daily exchange system. Only small amounts of fruits and vegetables containing phenylalanine 76–100 mg/100 g were eaten by this group of children, and so this will have constrained any adverse impact on blood phenylalanine. We also only studied children who generally eat smaller fruits and vegetable portion sizes compared with adults.

## 5. Conclusions

In this cohort of children with PKU, the preliminary data on the unlimited use of fruits and vegetables containing phenylalanine 76–100 mg/100 g showed no adverse impact on blood phenylalanine levels, but the portion sizes consumed were small and were equivalent to one 60 g portion/week. For this reason, the results need to be interpreted cautiously. It is important to continue to adhere to the PKU European Guidelines on the free use of fruits and vegetables for patients with classical PKU [3]. However, patients with milder forms of PKU and patients with higher tolerance or using adjunct pharmaceutical therapies may be able to tolerate the unrestricted use of these fruits and vegetables, facilitating more dietary freedom. Personalised medicine should be the core of our management, whereby the individual needs of patients are considered, reflecting their different phenotypes, phenylalanine tolerance and pharmaceutical treatments.

## Figures and Tables

**Table 1:** Participants’ characteristics and dietary prescription 6 months pre and post-trial.

Subject Number	Age at the Start of 6 Months Follow-Up (Years)	Sex	Mutations	Phenotype (Based on Genetics or Phenylalanine Tolerance)	Number of Exchanges Prescribed (1 g Protein = 50 mg of Phenylalanine) Pre vs. Post-Trial	Protein Equivalent Amount (g/day) from Protein Substitute (Pre and Post-Trial)	Protein Substitute Brand, Type and Size (Pre and Post-Trial)
1	10	F	Unknown (Molecular analysis not performed)	Classical PKU (based on diagnostic phenylalanine level)	4 vs. 5	65	PKU Air 15^®^ Vit (45 g PE) PKU Air 20^®^ Vit (20 g PE)
2	8	M	C1315+1G>A.p.? c.782G>A p(Arg261Gln)	Classical PKU	6.5 vs. 6.5	60	PKU Express 15^®^ Vit (45 g PE) PKU Air 15^®^ Vit (15 g PE)
3	12	M	Unknown	Mild PKU	25 vs. 27	60	PKU Sphere 20^®^ Vit (60 g PE)
4	13	F	c.194T>C p.(Ile65Thr) C.1066-11G>Ap.?	Mild PKU	14 vs. 14	60	PKU Sphere 20^®^ Vit (60 g PE)
5	9	F	C194t>c c.1222C>T p.(Arg408Trp)	Classical PKU	6 vs. 6	60	PKU Sphere 20^®^ Vit (60 g PE)
6	12	M	c. 1066-11 G>A p. ? c. 912+1 G>A p. ?	Classical PKU	7.5 vs. 7.5	80	PKU Sphere 20^®^ Vit (60 g PE) PKU Cooler 20^®^ Vit (20 g PE)
7	13	F	c.47_48del p.(Ser16*) c.1222C>T p.(Arg408Trp)	Classical PKU	4.5 vs. 4.5	80	PKU Sphere 20^®^ Vit (80 g PE)
8	9	M	c.1222C>T p.(Arg408Trp) c.1222C>T p.(Arg408Trp)	Classical PKU	3 vs. 3	80	PKU Sphere 20^®^ Vit (60 g PE) PKU Cooler 20^®^ Vit (20 g PE)
9	6	F	c.745C>T p.(Leu249Phe) c.1315+1G>A.p.?	Classical PKU	5.5 vs. 5.5	60	PKU Cooler 15^®^ Vit (60 g PE)
10	12	F	c.558_559del p.(Trp187Glyfs*12) c.558_559del p.(Trp187Glyfs*12)	Classical PKU	4 vs. 4	60	PKU Air 20^®^ Vit (40 g PE) PKU Sphere 20^®^ Vit (20 g PE)
11	12	F	c.782G>A p.(Arg261Gln) c.896T>G p.(Phe299Cys)	Classical PKU	6 vs. 7	60	PKU Lophlex 10^®^ Nu (60 g PE)
12	12	F	c.558_559del p.(Trp187Glyfs*12) c.558_559del p.(Trp187Glyfs*12)	Classical PKU	4 vs. 4	60	PKU Air 20^®^ Vit (40 g PE) PKU Sphere 20^®^ Vit (20 g PE)
13	11	F	c.782G>A p.(Arg261Gln) c.896T>G p.(Phe299Cys)	Classical PKU	5.5 vs. 6.5	60	PKU Lophlex 20^®^ Nu (60 g PE)
14	11	F	c.1042C>G p.(Leu348Val) c.1315+1G>A p.?	Classical PKU	6 vs. 6	70	PKU Cooler 20^®^ Vit (60 g PE) PKU Cooler 10^®^ Vit (10 g PE)
15	9	M	c.926C>T p.(Ala309Val) c.1103A>G p.(Glu368Gly)	Classical PKU	4 vs. 4	60	PKU Air 20^®^ Vit (40 g PE) PKU Sphere 20^®^ Vit (20 g PE)
16	10	F	c.782G>A p.(Arg261Gln) c.1222C>T p.(Arg408Trp)	Classical PKU	6 vs. 6	60	PKU Air 20^®^ Vit (60 g PE)

Abbreviations: PKU, phenylketonuria; PE, protein equivalent; F, female; M, male; Vit, Vitaflo International Limited; Nu, Nutricia Limited.

**Table 2:** Metabolic control of all participants pre-trial and during follow-up.

Metabolic Control	6 Months Pre-Trial	6 Months Follow-Up
**Median (range) phenylalanine levels (μmol/L)**	270 (50–760)	250 (20–750)
**Mean ± SD phenylalanine levels (μmol/L)**	284 ± 136	271 ± 142
**% levels <360 μmol/L**	74	75
**Median (range) tyrosine levels (μmol/L)**	50 (20–200)	50 (20–210)
**Total number of blood spots for all subjects**	334	348

Abbreviations: SD, standard deviation.

**Table 3:** Actual subject dietary intake during the 6-month follow-up.

	Month 1 *Mean* *(Range)*	Month 2 *Mean* *(Range)*	Month 3 *Mean* *(Range)*	Month 4 *Mean* *(Range)*	Month 5 *Mean* *(Range)*	Month 6 *Mean* *(Range)*	Total *Mean* *(Range)*
**Energy** **(kcal/day)**	1775 (1177–2862)	1779 (1127–2781)	1815 (1032–3029)	1783 (1151–2986)	1740 (1085–2714)	1779 (1167–2726)	1851 (1032–22,044)
**CHO** **(g/day)**	240 (130–437)	251 (151–461)	248 (135–497)	240 (130–452)	238 (138–461)	239 (132–423)	242 (130–497)
**CHO** **(% energy)**	54 (35–63)	56 (44–73)	55 (43–71)	54 (35–63)	55 (35–73)	54 (44–64)	55 (35–73)
**Total protein** **(g/day)**	76 (65–92)	76 (64–96)	77 (65–93)	76 (64–95)	76 (64–92)	76 (65–97)	76 (64–97)
**Total protein** **(% energy)**	18 (12–27)	18 (12–26)	18 (12–26)	18 (12–29)	18 (12–27)	17 (12–23)	18 (12–29)
**Fat** **(g/day)**	57 (27–97)	53 (26–104)	56 (17–104)	58 (30–113)	52 (25–117)	55 (28–104)	55 (17–117)
**Fat** **(% energy)**	29 (19–45)	27 (15–42)	27 (13–41)	28 (15–45)	27 (15–52)	29 (20–64)	27 (13–64)
**Natural protein** **intake (g/day)**	11.4 (5–31)	11.3 (4–35)	11.7 (5–33)	11.0 (4–34)	10.9 (4–31)	11.6 (4–37)	11 (4–37)
**Phenylalanine (mg/day)**	571 (250–1550)	560 (200–1750)	576 (250–1650)	553 (200–1700)	547 (200–1550)	574 (200–1850)	563 (200–1850)
**Phenylalanine (mg/day) from fruit/vegetables containing phenylalanine 76–100 mg/100 g food**	14 (0–100)	14 (0–100)	24 (0–100)	24 (0–146)	18 (0–100)	21 (0–103)	19 (0–146)

Abbreviations: CHO, carbohydrate.

**Table 4:** Monthly weight z-scores of participants during the study.

	Month 1 *Mean* *(±SD)*	Month 2 *Mean* *(±SD)*	Month 3 *Mean* *(±SD)*	Month 4 *Mean* *(±SD)*	Month 5 *Mean* *(±SD)*	Month 6 *Mean* *(±SD)*
**Weight** **z-score**	1.52 (±0.76)	1.55 (±0.78)	1.59 (±0.74)	1.68 (±0.68)	1.57 (±0.72)	1.60 (±0.73)

Abbreviations: SD, standard deviation.

## Data Availability

Not applicable.
